# Innate Lymphoid Cells Are the Rheostat of Immune Response in the Kidney

**DOI:** 10.1111/jcmm.70782

**Published:** 2025-08-13

**Authors:** Sensen Su, Lei Wang, Han Qin, Hui Yu, Siyuan Ma, Yueming He, Xin Chen, Zhanchuan Ma, Heyuan Wang, Huanfa Yi

**Affiliations:** ^1^ Central Laboratory The First Hospital of Jilin University Changchun China; ^2^ Department of Nephrology The First Hospital of Jilin University Changchun China; ^3^ Key Laboratory of Organ Regeneration and Transplantation Ministry of Education Changchun China; ^4^ Second Department of Gastrocolorectal Surgery Jilin Cancer Hospital Changchun China; ^5^ Department of Pediatric Cardiology, Children's Medical Center The First Hospital of Jilin University Changchun China; ^6^ Gastroenteric Medicine and Digestive Endoscopy Center The Second Hospital of Jilin University Changchun China; ^7^ Department of Endocrinology and Metabolism The First Hospital of Jilin University Changchun China

**Keywords:** acute kidney injury, chronic kidney disease, glomerulonephritis, innate lymphoid cells, renal fibrosis

## Abstract

Kidney disease ranks as the seventh most significant and the third fastest‐growing risk factor contributing to mortality globally. Innate lymphoid cells (ILCs) are tissue‐resident immune cells that lack antigen‐specific receptors and produce robust cytokines. ILCs play vital roles in infection, allergy, metabolic disorders, cancers, and tissue homeostasis. Recent studies have found that ILCs are manipulated for various kidney diseases. ILCs are classified into natural killer (NK) cells, ILC1s, ILC2s, ILC3s, and regulatory ILCs (ILCregs). We mainly discuss ILC1s, ILC2s, and ILC3s in kidney diseases. ILC2s and ILC3s are distributed along the renal vessels, and ILC3s are involved in the formation of ectopic lymphoid structures. ILCs secrete a variety of active cytokines, which can directly act on renal parenchymal cells or recruit other immune cells to affect kidney disease. Both acute kidney injury (AKI) and chronic kidney disease (CKD) are regulated by ILCs. ILC2s play a protective role in AKI and glomerulonephritis. Though ILC3s promote fibrosis in CKD, the roles of ILC2s in kidney fibrosis remain controversial. ILC1s and ILC3s promote glomerulonephritis. Kidney diseases will benefit from further studies focusing on the epigenetic/metabolic/neuron modulation and plasticity of ILCs.

## 1 Background

Kidney disease ranks as the seventh most significant and the third fastest‐growing risk factor contributing to mortality globally [1]. Both acute kidney disease (AKI) and chronic kidney disease (CKD) are complex clinical entities and are intricately governed by immune mechanisms. The immune system within the kidneys demonstrates a sophisticated and ever‐changing interaction between innate and adaptive responses, collaborating to detect pathogens, preserve tissue integrity, and modulate immune reactions [2]. The innate immune system of the kidneys functions as the primary defence mechanism [3, 4, 5]. While the adaptive immune system exhibits a specific immune response, immune homeostasis and imbalance play vital roles in various kidney diseases; however, the immune landscape remains unclear.

Innate lymphoid cells (ILCs) are newly identified tissue‐resident immune cells [6]. The barrier organs possess the most abundant ILCs, such as the pulmonary, the gut, and the skin. Parenchymal organs also constitute the sites where ILCs reside and exert their functions, such as the liver, the uterus, the kidney, etc. These cells present conserved traits among vertebrates, such as zebrafish, rodents, and humans, underscoring their paramount importance and indispensable nature [7]. While ILCs in different tissues demonstrate tissue‐specific behaviours [8].

ILCs are the innate counterparts of T cells. They lack antigen‐specific receptors and exhibit immediate responsiveness to environmental stimuli and high cytokine production [9]. The tissue‐dwelling characteristics of ILCs make them not only the fastest responders in immune reactions and the frontline of adaptive immunity but also pivotal in maintaining tissue homeostasis [9]. Emerging evidence has elucidated the roles of ILCs in barrier organs; comparable little is known in the kidney. ILCs secrete a variety of active cytokines, which can directly act on renal parenchymal cells or recruit other immune cells to affect kidney disease. In this review, we will discuss ILCs in kidney diseases and elucidate ILCs acting as the rheostat of kidney diseases.

## 2 Phenotypes of ILCs

**FIGURE 1:**
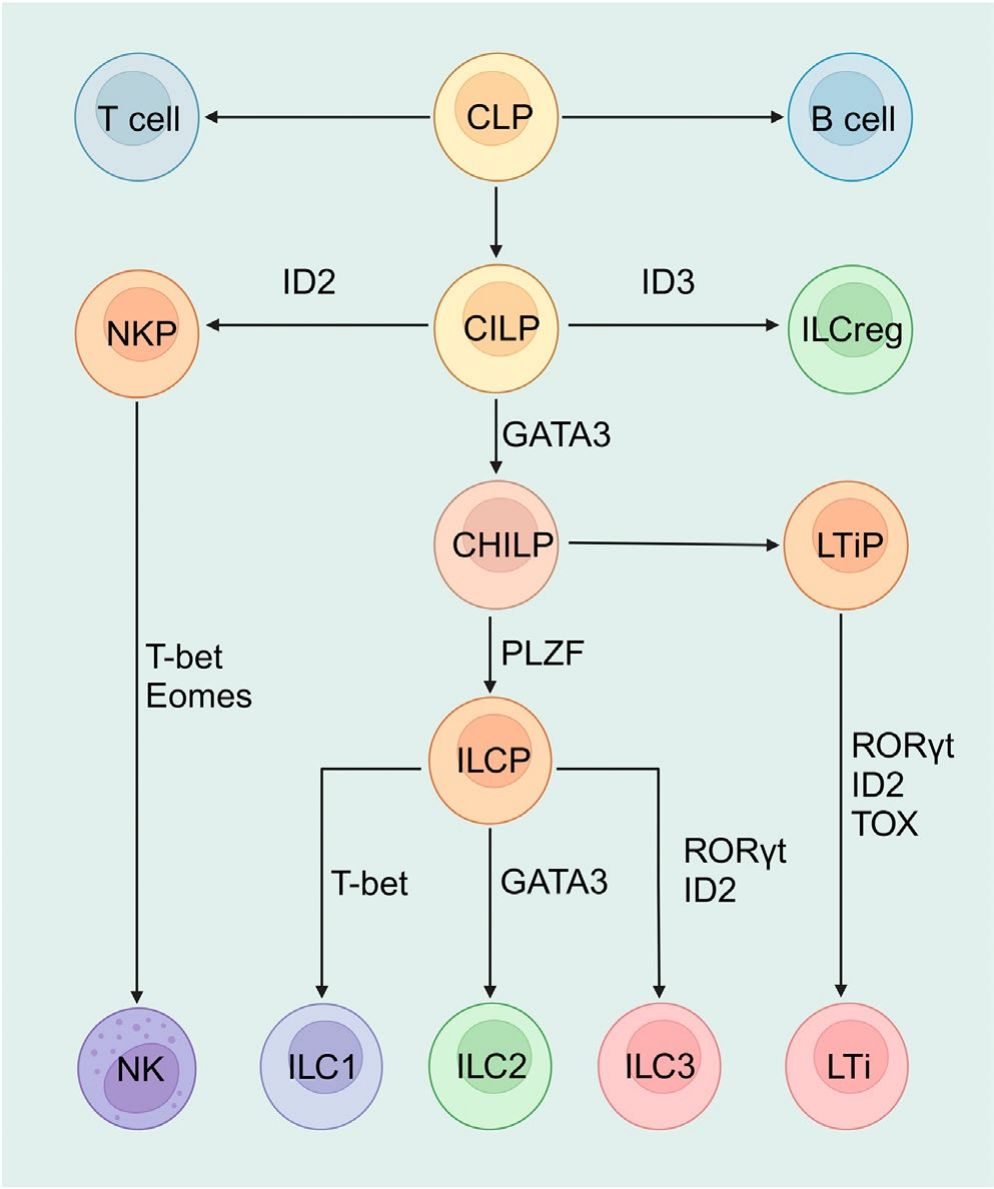
The development of ILCs. CLIP (common innate lymphoid progenitors) are derived from CLP (common lymphoid progenitors). CLIP can differentiate into NKP (NK precursors), which further mature into NK cells. Additionally, CLIP can directly differentiate into ILCreg cells. CLIP can also give rise to CHILP (common helper innate lymphoid progenitors), which subsequently differentiate into ILCP (innate lymphoid cell precursors). ILCPs further differentiate into ILC1s, ILC2s, and ILC3s. Moreover, CHILP can differentiate into LTiP (lymphoid tissue inducer progenitors), which further differentiate into LTi (lymphoid tissue inducers). Created with biorender (http://app.biorender.com).

**FIGURE 2:**
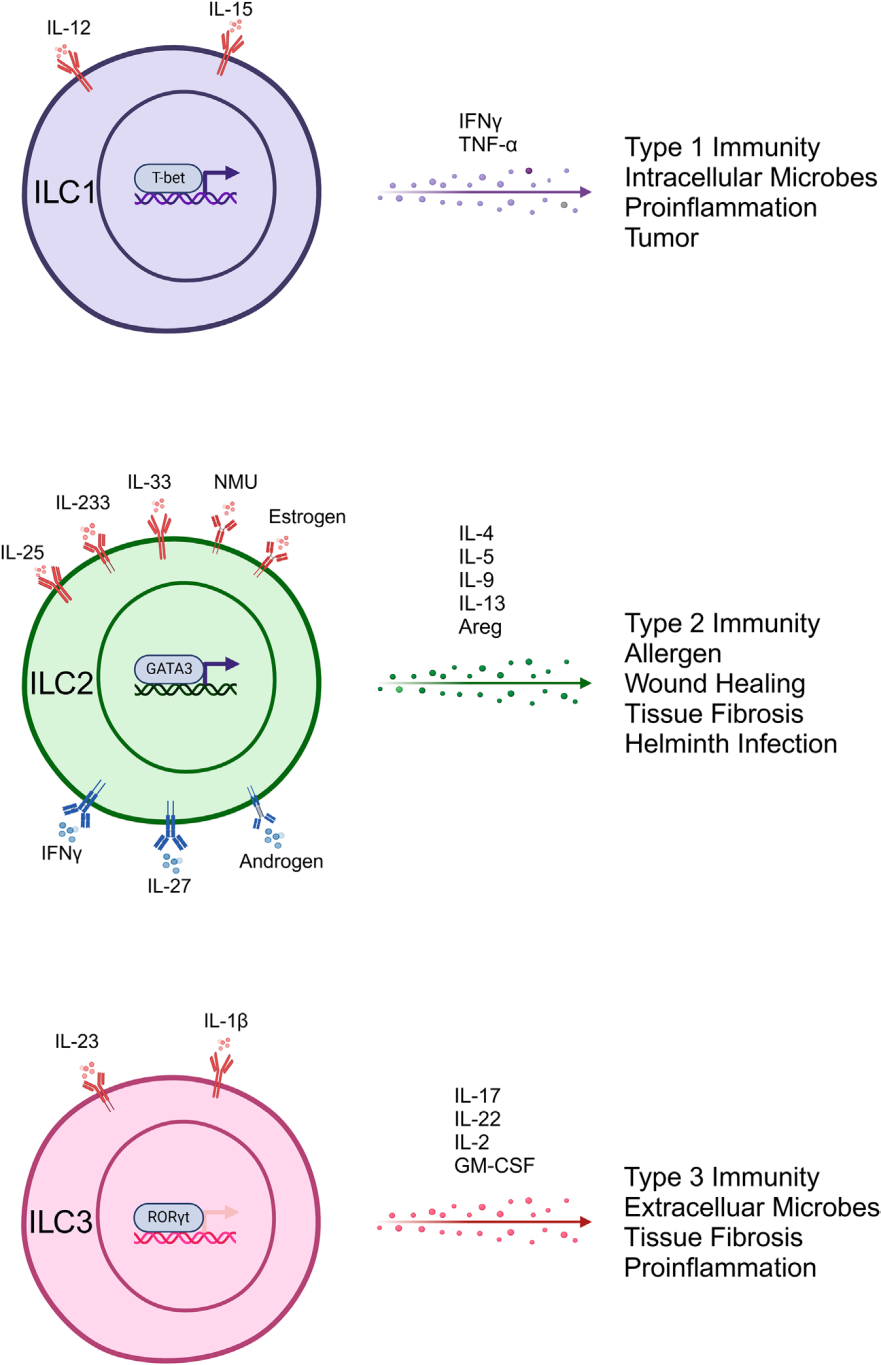
The modulation and function of ILC subtypes. The activating or inhibitory factors of the major ILC subtypes, their secreted bioactive molecules, and primary effector functions in renal diseases. Created with biorender (http://app.biorender.com).

ILCs are classified into Natural killer (NK) cells, ILC1s, ILC2s, ILC3s, regulatory ILCs (ILCregs), which correspond to the T‐cell subsets of CD8+ T cells, T helper cell 1, T helper cell 2, and T helper cell 17, and regulatory T cells [6]. ILC1s, ILC2s, and ILC3s share the common ancestor ILC progenitors (ILCP), while NK cells and ILCregs originate from common innate lymphoid progenitors (CILP). Lymphoid tissue‐inducer cells (LTi) have been recognised as a subtype of ILC3s [10]. The development of ILCs is shown in Figure 1. The role of NK cells in renal diseases has been well‐discussed recently elsewhere and will not be elaborated on here [11]. ILCs lack antigen‐specific receptors but are equipped with receptors that perceive various stimulatory signals. They act modulational functions either through active molecule excretion or direct cell–cell communication in inflammation, allergy, fibrosis, tumour, homeostasis, etc. [6, 12, 13]. The main features of different subsets are shown in Figure 2. The distribution of ILC subtypes in different tissues is completely different, which may be the result of adapting to the local environment and making the appropriate immune response in response to the stimulation from different tissues [8].

ILC1s depend on transcription factor T‐bet to develop from ILCP, and the main effector molecule is IFN‐gamma [14]. The precise phenotypic definition of ILC1s is still under debate due to the lack of a unique and reliable marker [15], and ILC1s are conversely heterogeneous and plastic [16, 17]. While some research groups compare NK cells with ILC1s, this grouping can be potentially misleading given their functional and developmental differences. NK cells originate from CILP other than ILCP, indicating the heterogenesis with ILC1s. ILC1s primarily respond to tissue inflammation and intracellular pathogens, such as viruses, bacteria, and certain parasites. ILC1s/IFN‐gamma protected the host from infections such as *Clostridium difficile* in the gut [18], cytomegalovirus in the liver [19], *mycobacterium tuberculosis* in the lung [20], etc. Growing evidence indicates that ILC1s are functionally implicated in diverse diseases, ranging from metabolic disorders to tissue regeneration and tumourigenesis. ILC1s/IFN‐gamma modulated the polarisation of proinflammatory macrophages in adipose tissue and disrupted metabolic homeostasis [21]. TGF‐beta1 was induced in ILC1s while cocultured with gut organoids, showing the ability to stiffen and soften the matrix [22]. ILC1s exhibited antitumour activity in a G‐protein‐coupled receptor 34 (GPR34)‐dependent manner [23]. Current evidence regarding ILC1s remains limited, warranting further investigation.

ILC2s are the most conserved subset among ILCs. GATA binding protein 3 (GATA3) is the key transcriptional factor for the development of ILC2s. ILC2s maintain conserved traits across different species and tissues, including GATA3 expression and secretion of type 2 cytokines, such as IL‐4, IL‐5, IL‐13, and amphiregulin, fulfilling the role of innate counterparts in type 2 immunity [24]. ILC2s are the longest‐lived immune cells, with a documented stable survival for up to 2 months in vivo [25, 26] and 12 months in vitro culture systems [27]. On the other hand, ILC2s exhibit plasticity in various tissues and under different surrounding environments [28]. ILC2s are activated by acute/chronic tissue damage and extracellular pathogens, including bacteria, parasites, and allergens, thus modulating metabolic homeostasis and tissue repair [29]. GATA‐3 silencing by tandem super‐enhancers (G3SE) knockout reduced ILC2s number in mice [24]. ILC2s constitutively express IL‐5, involving tissue homeostasis as well as reacting to stimuli [30]. The proliferation, activation, and inhibition of ILC2s are regulated by different cytokines, such as activating factors IL‐25, IL‐33, neuromedin U (NMU), and inhibiting factors IFN‐gamma and IL‐27, to achieve their diverse functions in tissues [31]. Following binding to their cognate receptors on ILC2s, IL‐25 and IL‐33 initiated intracellular signalling through NF‐kappa‐B and Mitogen‐Activated Protein Kinase (MAPK) pathways, ultimately driving GATA3‐mediated activation, cellular proliferation, and cytokine secretion [31]. Upon binding to Nmur1 on ILC2s, NMU initiated downstream signalling through extracellular signal‐regulated kinase (ERK) and calcium‐dependent calcineurin/nuclear factor of activated T cells (NFAT) pathways, resulting in ILC2 proliferation and activation [32]. The cytokines IFN‐gamma and IL‐27 mediated suppression of ILC2 function in a STAT1 transcription factor‐dependent manner, representing an intrinsic negative feedback pathway for ILC2 regulation [33]. As the primary responders in immune reactions, ILC2s play a crucial regulatory role in adaptive immunity. ILC2s recruited eosinophils and alternatively activated macrophages through IL‐5 and IL‐13, respectively [26, 34]. ILC2s mediate asthma and airway inflammation, adipose homeostasis, kidney disease, as well as tumours [35, 36, 37]. ILC2s exacerbated asthma by secreting cytokines IL‐5 and IL‐13 to promote airway hyperresponsiveness, whereas they conferred protective effects during pulmonary influenza infection by enhancing viral clearance and tissue repair [37]. IL‐33/ILC2s/alternatively activated macrophage (M2) promoted white adipose tissue beiging, improved lipid metabolism, alleviated insulin resistance, and reduced diabetes incidence, thereby playing a vital protective role in maintaining metabolic homeostasis [38]. ILC2s protect against various kidney diseases: IL‐25/ILC2s/IL‐5 axis reduces glomerulonephritis, while IL‐33/ILC2s/IL‐13 pathway alleviates ischaemic kidney injury [35]. Granzyme B (GZMB) secreted by human ILC2s directly lysed tumour cells by inducing pyroptosis and/or apoptosis [25]. The phagocytosis of ILC2s shows the antigen‐presenting capacity and new pathways to affect acquired immunity [39]. However, the role of ILC2s in the gut remains obscure.

ILC3s are differentiated by the transcription factor ROR‐gamma‐t and activated by local inflammation and extracellular microbes (commensals, pathogens, and fungi). Upon IL‐23 and IL‐1‐beta stimulation, they secrete IL‐17, IL‐22, or both. Intestinal ILC3s also secrete IL‐2 and GM‐CSF. ILC3s are abundant in the intestinal barrier and play an important role in intestinal homeostasis, bacterial infection, inflammatory bowel disease, food allergies, and cancers [40, 41]. Emerging evidence also indicates the important roles of ILC3s in other tissues, such as inflammation in the lung and bladder [42, 43]. Lymphoid tissue inducer (LTi) cells express the transcription factor ROR‐gamma‐t and induce the occurrence of secondary lymphoid tissues during embryogenesis. LTi cells are considered a subset of ILC3s rather than a separate one [10].

## 3 Distribution of ILCs in the Kidney

ILCs are mainly tissue‐resident cells, though some reside in circulation. They exert their immunomodulatory effects by interacting with parenchymal cells and adaptive immunity, thus acting as the rheostat of kidney immune responses. ILCs accounted for about 0.4%–1.0% of CD45+ immune cells in the kidneys of both mice and humans [26]. GATA‐3 expressing ILC2s constituted the most abundant subset in mice kidneys, approximately 80% of total ILCs [26]. While in humans, ILC2s and ILC3s constituted the most common subtypes [26]. The kidney‐resident ILC2s exhibited the most robust response to peritoneal injection of IL‐33 than other tissue‐resident ILC2s [26]. ILC2s were located both in glomeruli and tubulointerstitial compartments, around the vasculature under homeostatic conditions [26, 44]. ILC3s were distributed along renal blood vessels in the kidney tissue of mice 3 days after Unilateral Ureteral Obstruction (UUO) surgery [45]. In contrast, in the kidney tissue of MRL/lpr mice with lupus nephritis, ILC3s were predominantly localised within renal ectopic lymphoid structures (ELS) and exhibited closer spatial association with B lymphocytes rather than T lymphocytes [46]. Currently, there is a lack of studies on the precise localisation of ILC1s in renal tissues. The distribution of ILCs facilitates these cells to react immediately to various stimuli in the kidney.

## 4 ILCs in Kidney Diseases

ILC2s are the most studied ILC phenotype in kidney diseases while emerging evidence has indicated the important roles of other cell types. Although ILCs count small populations of the renal total CD45 cells, their abilities for high production of cytokines make ILCs the pivotal modulators in kidney diseases. The roles of ILCs in kidney diseases are shown in Table 1.

**TABLE 1:** ILCs in kidney diseases.

Diseases	References	Species	Inducing signals	Major findings
AKI	[47]	Mouse Human	IL‐2C	ILCregs proliferated by IL‐2C alleviate IRI‐AKI.
	[44]	Mouse	NA	The absence of ILC2s does not alter the severity of IRI‐AKI.
	[34]	Mouse Human	IL‐33	IL‐33/ILC2s alleviate IRI‐AKI. Amphiregulin secreted by ILC2s and M2 recruited by ILC2s provide protective effects.
	[48]	Mouse	IL‐233	IL‐233/ILC2s protect AKI induced by IRI, cisplatin, and doxorubicin.
	[49]	Mouse	IL‐25	IL‐25/ILC2/IL‐13/M2 alleviate IRI‐AKI
CKD				
Renal fibrosis	[50]	Mouse	IL‐33	ILC2 number decreased in UUO mice kidneys though IL‐33 is elevated. IL‐33 pretreated before UUO alleviates renal fibrosis. ROS and TGF‐β do not affect the proliferation of ILC2s.
	[51]	Mouse	IL‐33	HIF1α/ILC2s/M2 promotes renal fibrosis.
	[52]	Mouse	IL‐33	Both ILC2 number and cytokine production are decreased in adenine‐induced renal fibrosis mice. IL‐33/ILC2s attenuates adenine‐induced renal fibrosis.
	[53]	Human Mouse	CXCL16	ILC3s are enriched in the fibrotic iche of CKD patients. Intestinal ILC3s migrate to the kidney through CXCL16/CXCR6 axis and IL‐17A derived from ILC3s in PD‐1‐dependent manner promotes renal fibrosis.
MCD	[26]	Human Mouse	IL‐33	IL‐33/ILC2/eosinophils alleviate the kidney pathological change, ACR, and BUN of AN.
	[54]	Rat	IL‐33	IL‐33/ILC2/IL‐13 protects podocyte injury and alleviated proteinuria in both adriamycin and puromycin aminonucleoside‐induced MCD.
IgAN	[55]	Mouse	IL‐33	IL‐33/ILC2 exacerbates IgAN in BAFF‐transgenic mice.
Anti‐GBM	[56]	Rats Human	IL‐12/IL‐18	PPARα/ILC1/IFNγ exacerbate anti‐GBM nephritis.
LN	[57]	Mouse	IL‐33	ILC2s are decreased in MPL‐lpr mice through IFNγ and IL‐27 produced by CD4+ T cells and CD11b + monocytes. IL‐33/ILC2s alleviate proteinuria, inflammatory cell infiltration in kidneys, and glomerular size in MPL‐lpr mice.
	[46]	Human Mouse	CXCR6	ILC3s are increased in PBMC and kidneys in LN patients and MPL‐lpr mice, and promote autoimmune and nephritis. Intestinal originated ILC3s enrich in the kidney and activate B cells in a DLL1/Notch‐dependent manner.
	[58]	Mouse	NA	ILC3/IL‐22 aggregates nephritis in MPL‐lpr mice. IL‐22 binding to IL‐22R in kidney epithelial cells activates STAT3, thus enhances macrophage recruitment and promotes LN development.
DKD	[59]	Mouse	IL‐233	IL‐233 alleviates proteinuria and mesangial expansion in the kidneys. IL‐233 proliferates Treg and type 2 inflammatory cells such as ILC2, M2, Th2.
	[60]	Human	NA	ILC2/IL‐4 or IL‐13/TGF‐β1 exacerbate renal fibrosis in DKD, which is defined by proteinuria and eGFR.
chRCC	[61]	Mouse	IL‐15	ILC1s highly expressing granzyme‐A are positively associated with overall survival in chRCC. IL‐15 expression in chRCC is positively tracked with ILC1 response.

Abbreviations: AKI, acute kidney injury; Anti‐GBM, anti‐glomerular basement membrane; chRCC, chromophobe renal cell carcinoma; CKD, chronic kidney disease; DKD, diabetic kidney disease; IgAN, IgA nephrology; IRI, ischemia–reperfusion injury; LN, lupus nephritis; MCD, minimal change disease.

### 4.1 Acute Kidney Injury

AKI is a common disorder with high mortality and increased susceptibility to CKD [62]. Ischemia–reperfusion injury (IRI), nephrotoxic drugs, and sepsis are the top three causes of AKI [4]. Immune cell infiltration and activation, and immunomodulatory factors imbalance are key pathogenesis of AKI.

IRI is the most popular aetiology in AKI, in which ILCs play pivotal roles. Proliferative ILC2s by IL‐25, IL‐33, and IL‐233 injection before the IRI onset alleviated AKI [34, 48, 49]. Amphiregulin was the key effector molecule of the protective IL‐33/ILC2 axis, and amphiregulin silenced in ILC2s failed to exhibit the protective role in IRI [34]. Meanwhile, M2s were recruited by ILC2s through IL‐13 secretion and alleviated the apoptosis of tubular epithelial cells in IRI; this can be reversed by administration of an inhibitor of colony‐stimulating factor ‐‐GW2580 [34]. In clinical practice, IRI is often unpredictable and identified after the occurrence. The IL‐33/ILC2 axis worked even after IRI. IL‐33 and IL‐233 injection or ILC2s adoptive transfer after ischaemia surgery alleviated renal injury in mice [34, 48]. Another intervention point in IRI is the AKI transferring to CKD; the role of ILC2 in this process is unknown.

However, the role of the IL‐33/ST2 axis in renal IRI remains controversial [63, 64, 65]. Some studies have demonstrated that IL‐33 or ST2 knockout exerted a protective effect against IRI‐AKI, with potential pathogenic factors including CD4+ T cells and iNKT cells [63, 64]. Unlike the protective effect of the IL‐33/ST2 axis mediated through ILC2 expansion, these conflicting findings may be attributed to differences in IL‐33 dosage, timing of administration, and the animal models used [63, 64, 65]. Moreover, the reduction and deletion of ILC2s did not affect renal IRI in mice [44] and were consistent with the observation that patients lacking these cells after bone marrow reconstitution do not show increased susceptibility to specific diseases [66]. These seemingly contradictory results may emphasise the compensatory role of Th2 and other immune cells in ILC2 knockout mice [44]. Meanwhile, unlike most studies that assessed the severity of renal injury at 24 h after reperfusion, in the ILC2 reduced and deletion study, the time point for assessing renal injury was 7 days after reperfusion. However, the injured renal tubules started to repair on day 7, which may be another reason [34, 44, 48, 49, 67]. Even though controversies still exist, the protective ILC2 effects make this phenotype a promising therapeutic target in IRI. More elaborately designed experiments are needed to verify the roles of ILC2s in IRI.

Other phenotypes of ILCs have also been studied in IRI. Expansion of ILCregs by IL‐2/IL‐2 antibody complexes (IL‐2C) prevented IRI in Rag−/− mice [68]. The protection of cell therapy was associated with the reduction of neutrophil infiltration and induction of M2 [68]. Whether ILC1 and ILC3 affect IRI remains obscure. Th1 and Th17 promoted the pathogenesis of IRI‐AKI, which indicated the promising effects of ILC1s and ILC3s [69, 70]. Further studies are needed to elucidate the exact parts of these ILC phenotypes.

**FIGURE 3:**
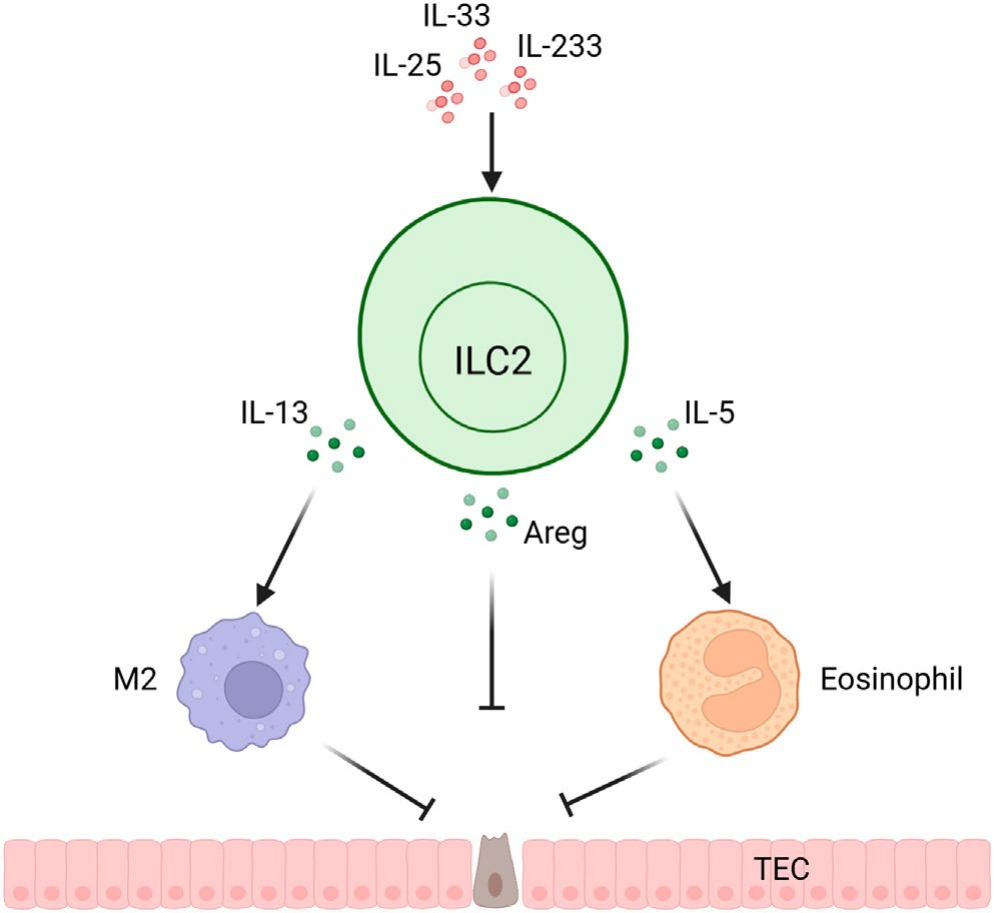
ILC2s in acute kidney injury. IL‐25, IL‐33, and IL‐233 promote the expansion and activation of ILC2s in the kidneys. These activated ILC2s secrete IL‐5, which recruits eosinophils, IL‐13, which attracts M2 macrophages, and Amphiregulin (Areg). Collectively, these factors help reduce tubular epithelial cell death, thereby providing protection against acute kidney injury. Created with biorender (http://app.biorender.com).

ILC2s also played protective roles in cisplatin and doxorubicin‐induced AKI, which indicated the protective effect of different etiologies in AKI [48]. Due to the inevitable onset of AKI, studies focusing on the repair and AKI‐CKD transition gained more and more attention. IL‐4, IL‐10, and IL‐13 are well‐known cytokines involved in the above pathogenesis, and ILC2s may have promising roles. The roles of ILC2s in AKI are shown in Figure 3.

### 4.2 Chronic Kidney Disease

#### 4.2.1 Renal Fibrosis

CKD refers to abnormalities of kidney structure or function for at least 3 months [71]. CKD affects 9.1% of the population globally and results in 1.2 million deaths every year [72]. Renal fibrosis is the common pathophysiological manifestation of CKD and leads to glomerulosclerosis, tubule atrophy, vascular rarefaction, and interstitial fibrosis [73]. Immune microenvironment disorder is one of the most important causes of renal fibrosis, in which ILCs play indispensable roles.

Circulating ILCs and ILC1s were increased in diabetic kidney disease (DKD) patients compared with normal controls, highlighting the possible roles of ILC1s in renal fibrosis. Further investigations are needed to elucidate the exact roles of ILC1s in renal fibrosis [74].

Though ILC2s exhibit profibrotic effects in various diseases such as the lung, skin, heart, and liver, their roles in renal fibrosis are controversial [75, 76, 77, 78]. The protective roles of ILC2s in renal fibrosis have been mechanistically demonstrated in the following context. IL‐33/ILC2s ameliorated adriamycin‐induced glomerulosclerosis through induction of eosinophils. Myeloid cells decreased, accompanied by less fibrosis, while eosinophils increased significantly. Meanwhile, Rag−/− mice treated with IL‐33 and adriamycin retained this beneficial effect; however, the deletion of eosinophils reversed the pathological and clinical characteristics [26]. IL‐233 potentiated ILC2s/Tregs and alleviated renal fibrosis in doxorubicin‐induced renal injury before and even after 2 weeks of injection of doxorubicin hydrochloride [79]. However, the underlying mechanism remained obscure. In adenine‐induced renal fibrosis mice, both the number and cytokine‐production function of ILC2s were suppressed. The proliferation of ILC2s induced by IL‐33 suppressed the transition of renal fibroblasts to myofibroblasts, thereby ameliorating renal fibrosis [80]. The number of ILC2s decreased in unilateral ureteral obstruction (UUO) mice kidneys, and IL‐33 pretreated before UUO could protect renal fibrosis. However, ILC2 number and function had no relation to ROS and TGF‐β, which were common pathogenic factors in UUO [81]. IL‐233 injection proliferated ILC2s and Treg and improved DKD in genetically obese OB mice. Proteinuria and mesangial expansion were alleviated, but renal fibrosis was not evaluated [59]. Since renal fibrosis is accompanied by DKD, we speculate that IL‐233/ILC2s have a protective effect on DKD.

In contrast to these anti‐fibrotic effects, compelling data also exist to support ILC2s as drivers of renal fibrosis. Hypoxia‐inducible factor‐prolyl hydroxylase (HIF‐PHD) inhibitors alleviated renal fibrosis through reduced phosphorylation of p38MAPK in ILC2s; thus, M2 polarisation followed by IL‐4/IL‐5/IL‐13 production was decreased, suggesting the pathogenesis role of ILC2/cytokines/M2 in renal fibrosis [82]. In B‐cell activating factor (BAFF)‐transgenic mice, IL‐33 exacerbated glomerulosclerosis in an ILC2‐dependent manner [55]. Circulating ILC2s were significantly elevated in patients with DKD compared with normal controls and were associated with worse renal outcomes, manifested by elevated proteinuria and diminished estimated glomerular filtration rate (eGFR) [60]. IL‐4, IL‐5, and IL‐13 were elevated accordingly, and HK‐2 treated with these cytokines had higher expression of TGFβ, fibronectin, and collagen 1 [60].

These divergent findings may be attributed to variations across disease models, temporal dynamics during disease progression, and spatial heterogeneity of ILC2 localisation (circulating versus kidney resident); more well‐designed experiments are needed to confirm the exact roles of ILC2s in renal fibrosis in various disease circumstances.

**FIGURE 4:**
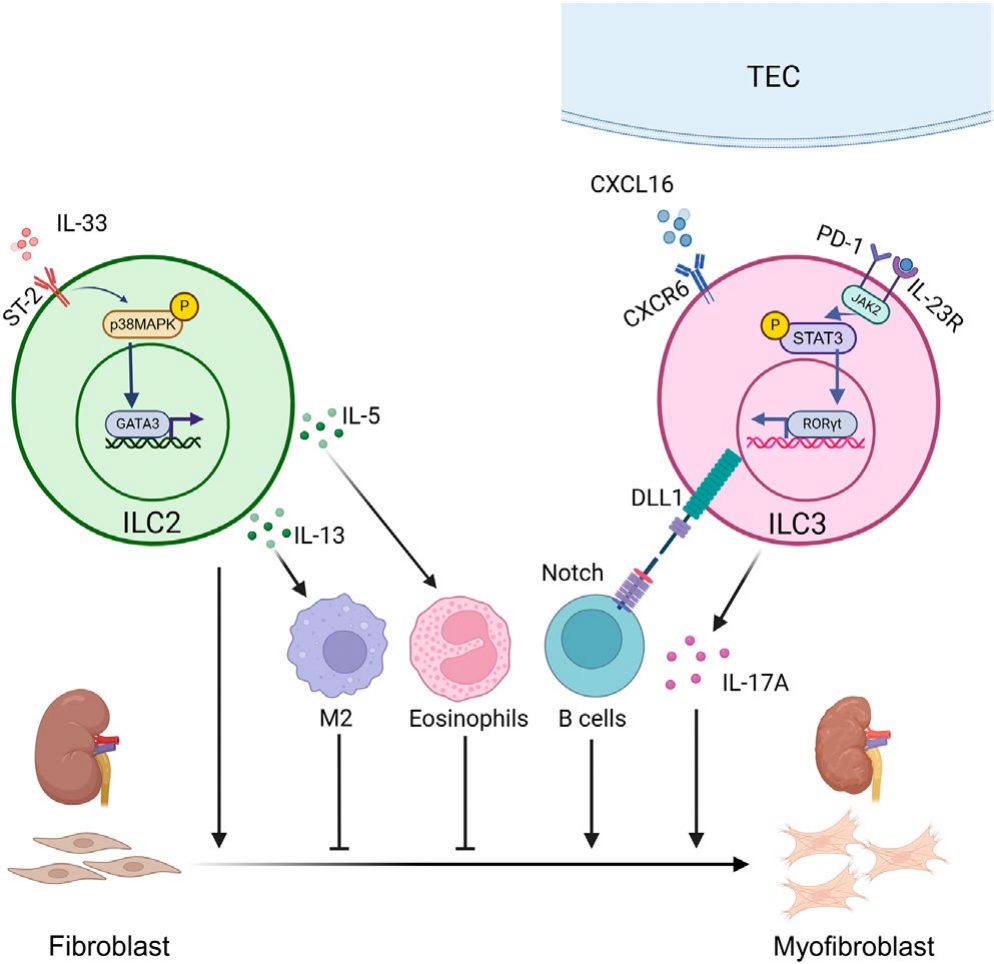
ILCs in renal fibrosis. ILC2s secrete IL‐5 to recruit eosinophils and IL‐13 to recruit M2 macrophages, both of which inhibit renal fibrosis. However, some studies suggest that ILC2s may also promote renal fibrosis, although the underlying mechanisms remain unclear. Additionally, tubular cells secrete CXCL16, which acts on intestinal ILC3s via CXCR6, leading to the secretion of IL‐17A and subsequently promoting renal fibrosis. ILC3s can also recruit B cells, further facilitating the progression of renal fibrosis. Created with biorender (http://app.biorender.com).

Renal fibrosis is closely related to renal dysfunction. Circulating ILC3s (cILC3s) decreased in patients with renal dysfunction and positively correlated with eGFR, which indicated the underlying effects of ILC3s in renal fibrosis [83]. This changed cILC3 number may be explained by a recent study. ILC3s were enriched in the fibrotic niche in human and mouse kidneys and produced IL‐17A to promote renal fibrosis in a PD‐1‐dependent manner [45]. Interestingly, ILC3s enriching in the fibrotic kidneys migrated from intestinal ILC3s through the CXCL16/CXCR6 axis rather than proliferating from kidney‐resident ILC3s. Accordingly, ILC3s were decreased in the intestinal tissue [45]. ILC3s were increased in PBMC and kidneys in lupus nephritis (LN) patients and MPL‐lpr mice (genetic LN model) and positively correlated with glomerular sclerosis. B cells activated by ILC3s in a DLL1/Notch‐dependent manner were the pathogenesis of intestinal‐originated ILC3s in LN [46]. These studies confirm the crosstalk between organs of ILC3s and clarify the innovative working patterns of ILC3s. The roles of ILCs in renal fibrosis are shown in Figure 4.

#### 4.2.2 Minimal Change Disease

Glomerulonephritis encompasses multiple immune‐mediated disorders with inflammation in the glomerulus. ILCs act as the first line in inflammation and are pivotal immune cells exhibiting rheostat in both primary and secondary glomerulonephritis. Minimal change disease (MCD) is the common phenotype of nephrotic syndrome, especially in children. Destruction of glomerular basement membrane (GBM) integrity following podocyte injury is the key process in MCD. IL‐33 injection following adriamycin and puromycin aminonucleoside‐induced MCD resulted in an upregulation of ILC2 markers, including GATA‐3 and IL‐13/IL‐5, in rat kidneys. Concurrently, kidney injury indicators such as proteinuria, desmin expression, inflammatory index, and renal pathological alterations were significantly reduced [84]. The protective effects of IL‐33 lasted 60 days, indicating the protective roles of IL‐33 in MCD. Eosinophils were reported to be essential for the protective roles of ILC2s in the IL‐33‐treated adriamycin‐induced glomerulosclerosis [26]. However, eosinophils have always been considered pathogenic, and further studies are needed. IL‐233 also reversed adriamycin‐induced MCD kidney structure and function in BALB/cJ mice, and ILC2s and Tregs were elevated significantly, suggesting the beneficial effects of ILC2s in this disease model [79].

#### 4.2.3 IgA Nephropathy

IL‐33/ILC2s exacerbated the renal function decline and pathological injury in BAFF‐transgenic IgAN mice. Adoptive transfer of splenocytes from IL‐33‐treated Rag1−/− mice into BAFF‐transgenic mice exacerbated IgAN deposition and glomerulosclerosis and increased proteinuria and serum creatinine. Interestingly, CD4‐CD19+ B cells in the spleen increased significantly in BAFF‐transgenic mice compared to wild types and further increased in IL‐33‐treated BAFF‐transgenic mice than in BAFF‐transgenic mice. CD4‐CD19+ B cells in the kidneys were increased in the Rag mice treated with splenocytes from IL‐33‐treated BAFF‐transgenic mice than in BAFF‐transgenic mice and were reversed by ILC2s deletion by anti‐CD90.2 [55]. There may be an underlying connection between ILC2 and B cells, which needs to be further clarified.

#### 4.2.4 Anti‐GBM Glomerulonephritis

ILC1s were involved in anti‐glomerular basement membrane (GBM) glomerulonephritis, especially in the early stages, prior to crescent formation. The PPARα agonists could inhibit the enrichment and function of ILC1s [56].

#### 4.2.5 Lupus Nephritis

ILC2s were decreased in the kidney in MPL‐lpr mice through the negative modulation of IFN‐gamma and IL‐27 produced by CD4+ T cells and CD11b + monocytes. IL‐33/ILC2s alleviated proteinuria, inflammatory cell infiltration in kidneys, and glomerular size in MPL‐lpr mice [57]. In LN patients, circulating ILCs, especially ILC1s were elevated, and both ILCs and ILC1s were positively correlated with the severity of LN [74]. However, ILC3salso increased in PBMC and kidneys in LN patients and MPL‐lpr mice. ILC3s were positively correlated with lupus activity indexes such as ANA, dsDNA, proteinuria, and kidney pathological lesions such as crescent and glomerular sclerosis. Adoptive transfer of ILC3s to MPL‐lpr mice accelerated autoimmune and nephritis development. Mechanically, ILC3s promoted LN through the proliferation and differentiation of B cells in a DLL1/Notch‐dependent manner [46]. IL‐22 derived from ILC3s was the main inflammatory molecule in MPL‐lpr mice. IL‐22 binded to IL‐22R in kidney epithelial cells and activated STAT3, thus promoting cytokines excretion such as CXCL10 and CCL2. These cytokines recruited macrophages to the kidney and aggregated LN [58].

#### 4.2.6 Diabetic Kidney Disease

ILC2s may be active modulators in DKD. Circulating ILC2s were elevated in DKD patients compared with normal controls and positively correlated with the severity of DKD defined by proteinuria and eGFR [60]. Circulating ILC2s as well as IL‐5 and IL‐13 were elevated in DKD patients with maintenance haemodialysis compared with healthy volunteers and newly diagnosed diabetic mellitus patients [85]. However, studies in the kidney resident ILC2s in mice showed different clues. IL‐233 reversed the progression of type 2 diabetes (T2D), onset of proteinuria, and mesangial expansion in younger OB mice at 5 weeks of age. Even after the onset of T2D and DKD, at 10 weeks of age, IL‐233 could improve blood glucose and proteinuria. The proliferation of Treg, ILC2s, and M2 might be effector cells in this process [59]. The controversial conclusions regarding ILC2s in DKD research may stem from their distinct tissue localisation or varying disease stages. In contrast, ILC1s and ILC3s in DKD remain scarce. More rigorously designed studies are needed to clarify the regulatory roles of ILCs in DKD.

Growing evidence underscores the indispensable roles of ILCs in glomerulopathy. They function as a rheostat in various inflammatory and reparative processes within the kidney. Targeting ILCs presents a promising strategy for resolving nephritis.

### 4.3 Renal Cell Carcinoma

Renal cell carcinoma (RCC) accounts for 2%–3% of total malignancies. Chromophobe RCC (chRCC) is the third most common RCC. Abundant infiltration of granzyme A‐expressing intraepithelial ILC1s was found to enrich the tumour of chRCC patients and was positively associated with overall survival. Interleukin‐15 (IL‐15) promoted ILC1 granzyme A expression and cytotoxicity, and IL‐15 expression in chRCC tumour tissue positively tracked with the ILC1 response [61].

Though other ILC subtypes prove modulatory roles in tumours, little is known about their effects on RCC [25, 86, 87]. Further investigations will clarify the underlying roles and mechanisms.

## 5 Prospectives

Emerging studies have deepened our understanding of ILCs in kidney diseases. As the roles of these rare cells are recognised, further research is needed to explore their mechanisms and potential involvement in the pathogenesis, progression, and treatment of kidney diseases.

The development and plasticity of ILCs are pivotal. Epigenetic modulations are intricately linked with transcriptional regulations, influencing the development, plasticity, and function of ILCs [88]. H3K4me3 had different enrichment landscapes in ILC1 (1268 enhancers), ILC2 (5934 enhancers), and ILC3 (4998 enhancers), indicating regulation of subtype‐unique gene expression [89]. However, the epigenetic regulation of ILCs remains to be explored, and the emergence of new technologies such as spatial Assay for Transposase‐Accessible Chromatin using sequencing (ATAC‐seq) technologies will facilitate our understanding of this field.

The sympathetic innervation covered all parts of the renal vasculature, most densely in afferent arterioles [90]. Besides, sensory nerves in the kidney were mainly in the pelvic region. ILCs were equipped with receptors for neuronal signals and neurons expressing receptors for ILC cytokines; they acted in bidirectional interactions [91]. NMU expressed from mucosal neurons was demonstrated to be an active modulator of ILC2s, and impairment of the NMU‐NMUr1 axis from ILC2s mitigated type 2 immune response in worm infection and allergic inflammation [32, 92]. Dopamine impaired mitochondrial oxidative phosphorylation (OXPHOS) in ILC2s and restrained lung inflammation through dopamine receptors Drd1 [93]. However, the exact roles of the nervous system and ILCs in the kidneys remain unclear and require further research, thus warranting future clinical applications of innovative therapy.

Immune dysregulation and metabolic disturbances are common features in various kidney diseases [94]. ILCs are characterised by rapid response to environmental stimuli, high production of cytokines, and plasticity, suggesting that ILCs are highly metabolised immune cells. As the integral adaptive parts of ILCs, metabolic changes ran through the lifespan of T cells [95]. Emerging evidence has revealed the metabolic landscape of ILCs [96]. Naive ILC2s fuelled OXPHOS for maintenance with sufficient branched‐chain amino acids and arginine, while the number of ILC2s was reduced in individuals with impaired mitochondria [97]. Aerobic glycolysis was fuelled to serve energy for the proliferation and cytokine excretion of ILC2s in pulmonary allergic inflammation, which was impaired by arginase‐1 deletion in mice [98]. Fatty acid oxidation (FAO) was vital for the energy supply of ILC2s in the context of helminth infection and malnutrition, such as vitamin A deficiency [99]. The activation of ILC3s depended on glycolysis modulated by mTOR complex 1 and HIF1α, and mitochondrial reactive oxygen species (ROS) for stabilising HIF‐1‐alpha [100]. Intestinal dysbiosis exacerbated Clostridioides difficile infection in mice by impairing HIF‐1‐alpha‐dependent IL‐22 production in ILC3s [101]. The sophisticated energy supply and regulatory network of ILCs are in the preliminary stage and difficult to track, especially in kidney diseases. The metabolic regulation of immune cells often intersects with parenchymal cell disorders and dysbacteriosis, complicating its mechanism and posing challenges for research transformation. Immunometabolism underpins ILC cell differentiation and function and offers a means to interfere with kidney diseases.

ILCs migrated inter‐organs during various pathological circumstances and complement adaptive immunity. ILC2 migrated gut—lung [102, 103], lung–gut [104], lung–liver [105] during inflammation. ILC3 can migrate gut–heart [106] during inflammation, and gut–kidney during kidney fibrosis [45, 46]. Intestinal glucocorticoids have been successful in treating IgAN, suggesting the existence of a gut–kidney axis [107]. In renal fibrosis, ILC3 migrated from the intestine to the kidney and aggravated renal fibrosis, which was a powerful annotation of the gut–kidney axis [45]. Further studies are needed to clarify the migration of the tissue‐resident ILCs activated upon local stimuli that contribute to systemic immune responses.

cILCs are the counterparts of ILCs that are located in peripheral blood circulation. cILCs are not the simple contrast of tissue‐resident ILCs. They may act in distinct functions. The proportion of ILCs in immune cells and ILC subgroups changed in CKD, and varied among different diseases [74]. cILCs decreased with age, with the highest proportion in umbilical cord blood [108]. The exact roles of cILCs remain obscure and need further investigation.

Recommended research directions for ILCs in kidney diseases are summarised in Table 2. New technologies in single‐cell RNA sequencing (scRNA‐seq) such as scATAC‐seq, scMNase‐seq, and spatial ATAC‐seq, along with kidney organoid models, will offer novel insights into the development, differentiation, inflammatory regulation, and interactions of ILCs with parenchymal cells in the kidney.

**TABLE 2:** Recommended research directions for ILCs in kidney diseases.

Key knowledge gaps	Recommended research directions
The development and plasticity of ILCs in kidney remain obscure	Epigenetic modulations of development and plasticity of ILCs
Despite renal sympathetic innervation and ILCs' neuroregulatory receptors, their interplay remains unclear	Bidirectional neuroimmune interactions between the sympathetic nervous system and ILCs in the pathogenesis, development and therapy of renal disease
ILCs immunometabolic regulation in kidney disease remains mechanistically undefined	Modulating ILC differentiation and function through immunometabolism in renal pathology
Mechanisms of ILC interorgan migration and systemic inflammatory roles require elucidation	Migration mechanisms of locally activated ILCs and their roles in systemic inflammation
The proportion of cILCs varies across diseases, yet their precise roles remain elusive	Elucidating the functional mechanisms of cILCs in disease pathogenesis

Abbreviations: cILCs, circulating ILCs; ILCs, innate lymphoid cells.

## 6 Conclusion

ILCs are key immune cells resident in the kidney. Studies have demonstrated that they play crucial roles in various kidney diseases, including AKI, CKD, and various glomerular diseases. Due to the tissue‐resident characteristics, these cells are typically involved in the early stages of disease and continue to contribute to disease progression by activating adaptive immunity and regulating parenchymal cells, such as renal tubular epithelial cells. ILCs are also indispensable for maintaining tissue homeostasis and promoting tissue repair. Consequently, ILCs serve as a rheostat for the immune response in kidney disease, and targeting ILCs represents a promising therapeutic approach for kidney diseases.

## Author Contributions

**Sensen Su:** conceptualization (equal), visualization (lead), writing – original draft (lead). **Lei Wang:** conceptualization (equal), investigation (equal), writing – original draft (supporting). **Han Qin:** writing – original draft (supporting). **Hui Yu:** writing – review and editing (supporting). **Siyuan Ma:** writing – review and editing (supporting). **Yueming He:** writing – review and editing (supporting). **Xin Chen:** writing – review and editing (supporting). **Zhanchuan Ma:** writing – review and editing (supporting). **Heyuan Wang:** conceptualization (equal), visualization (supporting), writing – review and editing (lead). **Huanfa Yi:** conceptualization (equal), writing – review and editing (lead).

## Conflicts of Interest

The authors confirm that there are no conflicts of interest.

## Data Availability

Data sharing not applicable to this article as no datasets were generated or analysed during the current study.
